# Palpable Breast Lump Triage by Minimally Trained Operators in Mexico Using Computer-Assisted Diagnosis and Low-Cost Ultrasound

**DOI:** 10.1200/JGO.17.00222

**Published:** 2018-08-29

**Authors:** Susan M. Love, Wendie A. Berg, Christine Podilchuk, Ana Lilia López Aldrete, Aarón Patricio Gaxiola Mascareño, Krishnamohan Pathicherikollamparambil, Ananth Sankarasubramanian, Leah Eshraghi, Richard Mammone

**Affiliations:** **Susan M. Love** and **Leah Eshraghi**, Dr Susan Love Research Foundation, Encino, CA; **Wendie A. Berg**, Magee-Womens Hospital, University of Pittsburgh School of Medicine, Pittsburgh, PA; **Christine Podilchuk**, **Krishnamohan Pathicherikollamparambil**, **Ananth Sankarasubramanian**, and **Richard Mammone**, AI Strategy, Warren, NJ; **Richard Mammone**, Rutgers University, New Brunswick, NJ; and **Ana Lilia López Aldrete** and **Aarón Patricio Gaxiola Mascareño**, Instituto de Seguridad y Servicios Sociales de los Trabajadores del Estado Hospital Regional Valentin Gomez Farias, Jalisco, Mexico.

## Abstract

**Purpose:**

In low- to middle-income countries (LMICs), most breast cancers present as palpable lumps; however, most palpable lumps are benign. We have developed artificial intelligence–based computer-assisted diagnosis (CADx) for an existing low-cost portable ultrasound system to triage which lumps need further evaluation and which are clearly benign. This pilot study was conducted to demonstrate that this approach can be successfully used by minimally trained health care workers in an LMIC country.

**Patients and Methods:**

We recruited and trained three nonradiologist health care workers to participate in an institutional review board–approved, Health Insurance Portability and Accountability Act–compliant pilot study in Jalisco, Mexico, to determine whether they could use portable ultrasound (GE Vscan Dual Probe) to acquire images of palpable breast lumps of adequate quality for accurate computer analysis. Images from 32 women with 32 breast masses were then analyzed with a triage-CADx system, generating an output of benign or suspicious (biopsy recommended). Triage-CADx outputs were compared with radiologist readings.

**Results:**

The nonradiologists were able to acquire adequate images. Triage by the CADx software was as accurate as assessment by specialist radiologists, with two (100%) of two cancers considered suspicious and 30 (100%) of 30 benign lesions classified as benign.

**Conclusion:**

A portable ultrasound system with CADx software can be successfully used by first-level health care workers to triage palpable breast lumps. These results open up the possibility of implementing practical, cost-effective triage of palpable breast lumps, ensuring that scarce resources can be dedicated to suspicious lesions requiring further workup.

## INTRODUCTION

Breast cancer is the most common cause of death resulting from cancer among women worldwide, and the numbers are disproportionately high for women in developing countries.^1-3^ The percentage of women with breast cancer who die as a result of the disease is three times higher in low- and middle-income countries (LMICs) than in high-income countries.

In LMICs, 23% of new breast cancer cases occur among women age 15 to 49 years, compared with 10% of new breast cancer cases in high-income countries.^4,5^ Screening mammography is not readily available or appropriate in many LMICs and is less effective in premenopausal women with dense breasts. Most cancers therefore present as palpable masses^4,5^ identified either by patients or through clinical breast examinations. In a study from India, 85% of palpable masses in premenopausal women proved to be benign on fine-needle aspiration.^6^ Ultrasonography (US) is widely used in the management of palpable breast masses^7^; it is nonionizing, and it can be affordable. Although portable US systems are available,^8^ interpretation has generally been performed by experienced operators, usually radiologists or breast surgeons.

Computer-aided detection (CADe) and computer-aided diagnosis (CADx) systems have been developed for breast cancer; however, most research has focused on detection with mammography and diagnosis using high-end cart-based US, automated whole-breast US,^9^ or magnetic resonance imaging.^10^ All require experienced radiologists to interpret the images and use the CADe results as an aid for detection.^11-16^ No current CADe or CADx systems focus on breast cancer image analysis and diagnosis using low-cost portable US devices such as a triage-CADx system to distinguish those patients who need immediate additional medical attention and those who can be observed.

The triage-CADx system is based on convolutional neural networks (CNNs) with transfer learning. This architecture has been shown to provide state-of-the-art performance for CADx of lesions on mammograms^17^ as well as for CADe of thoraco-abdominal lymph node and interstitial lung disease classification.^18^

We previously showed the effectiveness of an earlier triage-CADx system using images from the American College of Radiology Imaging Network 6666 screening breast US study^19,20^ and also using collected and anonymized images from Magee-Womens Hospital of the University of Pittsburgh Medical Center.^21^ Although we were able to demonstrate excellent triage performance as assessed by area under the receiver operating characteristic curve (AUC), these results were based on images obtained with high-end cart-based US systems typically available in high-resource settings, and trained radiologists or technologists specializing in breast US imaging captured the images.

The purpose of this study was to demonstrate that nonradiologist health care workers in an LMIC setting can use a portable US system to acquire images of palpable breast lumps comparable to those obtained by trained radiologists and equally adequate for accurate triage with CADx.

## PATIENTS AND METHODS

### Triage-CADx System

**Fig 1:**
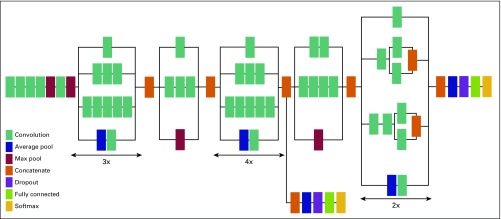
Inception-v3 convolutional neural network architecture used for breast cancer triage.

The triage-CADx system (AI Strategy, Warren, NJ) has been developed to operate as standalone software that can be run on a Windows 7.0 or higher operating system. The deep-learning CNN model used for this study is Inception-v3 using TensorFlow (www.tensorflow.org), and the architecture is shown in Figure 1. Because of limited breast US images for training, transfer learning was used to train and validate the classifier using curated data obtained from phase I of this study, including American College of Radiology Imaging Network data,^19,20^ anonymized images from Magee-Womens Hospital of University of Pittsburgh Medical Center,^21^ and images collected from University of Southern California and University of California Los Angeles during phase I. This model was then tested on the images collected in Mexico by both the nonradiologist health care workers and the trained radiologist. The model is compliant with digital imaging and communications in medicine (DICOM) and can process US images stored as DICOM files on a picture archiving computer system or US imaging device. It can also process images stored in standard image formats such as JPEG, TIF, or PNG.

**Fig 2:**
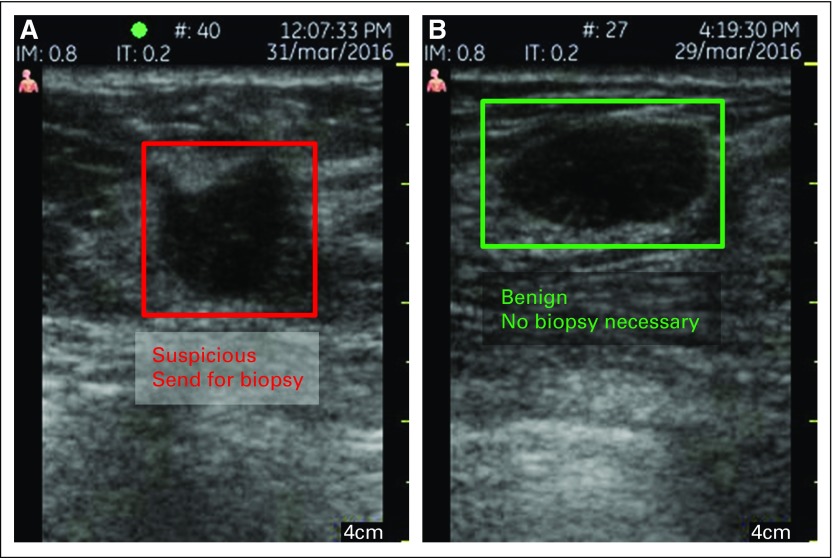
Output of the triage–computer-assisted diagnosis system, which correctly identified a (A) invasive ductal cancer and (B) fibroadenoma.

The output of the triage-CADx system is shown in Figure 2 using a standard DICOM viewer. The triage system can operate on one image, orthogonal views, or multiple views. Although previous results showed that taking the average score from multiple views provided the best overall results in terms of sensitivity, specificity, and AUC, this new CNN architecture was shown to provide the best performance using the maximum score from all the views. The deep-learning CNN on the basis of an Inception-v3 model has been trained to distinguish the following outcomes: red, suspicious (biopsy recommended) and green, benign (no further action necessary). The output of the CADx classifier produces a score on a scale from 0 to 1, where the highest scores correspond to suspicious lesions recommended for biopsy and the lowest scores correspond to the least suspicious lesions or those most likely to be benign.

### Patients and Setting

**Fig 3:**
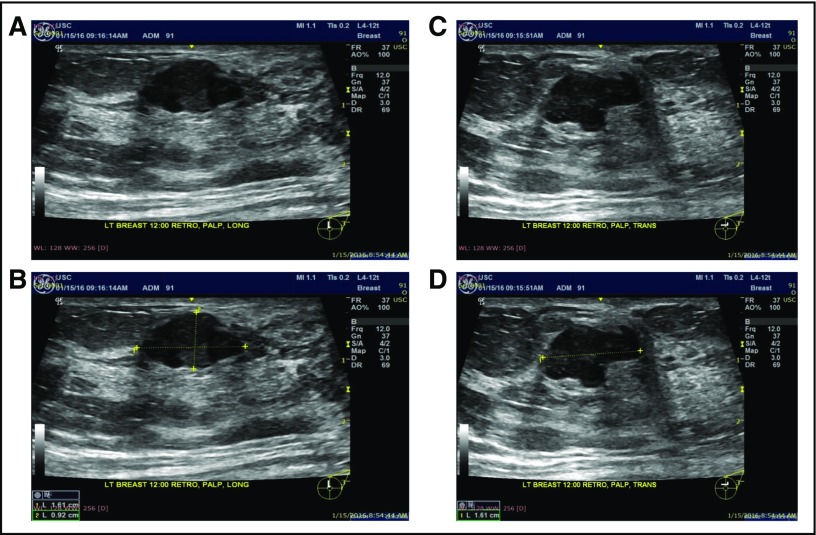
Training material describing how to capture orthogonal views with and without calipers. (A-D) Four images should be saved per woman. First, transducer is put along the longest part of the lump, and the picture is saved (A) before and (B) after measurement. Second, transducer is put along the shorter part of the lump, and the picture is saved (C) before and (D) after measurement.

The study was institutional review board approved and Health Insurance Portability and Accountability Act compliant. It was conducted in a resource-limited setting (Instituto de Seguridad y Servicios Sociales de los Trabajadores del Estado Hospital Regional Valentin Gomez Farias, Jalisco, Mexico) in March 2016 using a low-cost portable US device^8^ (GE Vscan Dual Probe; General Electric Medical Systems, Waukesha, WI) equipped with an 8-MHz linear array transducer. Three minimally trained health care workers with no prior experience in US imaging performed image acquisition. The three trainees participating in the study were chosen to represent first-level health care workers (those who are comfortable with patients but do not have any radiology experience) and included a first-year medical student, a surgical nurse, and a gynecologic intern. The operators were trained for approximately 30 minutes by the lead investigator (S.M.L.), using a PowerPoint presentation. They were taught to ask each woman to point out the palpable mass and then draw a line with a marker across the longest portion. A second line was then drawn perpendicular to the first. They were walked through using the GE Vscan Dual Probe device and shown how to take orthogonal views of the lump with and without caliper measurements. Figure 3 illustrates the training material for taking orthogonal views. The instructions were translated into Spanish.

Operators obtained two orthogonal views using the low-cost study machine (GE Vscan Dual Probe) and also using the in-house machine (HD11XE; Philips, Eindhover, the Netherlands) with L12-5 linear array probe. Three scenarios were examined: one, triage-CADx performance using in-house US device (HD11XE, equipped with L12-5 linear array probe) and radiologist; two, triage-CADx performance using low-cost portable GE Vscan Dual Probe device with 8-MHz linear probe scanned by a radiologist; and three, triage-CADx performance using low-cost portable GE Vscan Dual Probe device with 8-MHz linear probe scanned by trainee.

We recruited women with at least one self- or physician-identified palpable breast mass who were age ≥ 18 years presenting to a government hospital in Jalisco, Mexico, for evaluation in March 2016. Eligible participants had no history of breast surgery (eg, breast implant, reduction, cancer surgery) at the site of the palpable abnormality that would interfere with the intended field of view. Additionally, participants could not have existing mastitis or other signs of inflammation of the breast. All participants were informed about the study and consented in Spanish before examination. The hospital radiologist (A.P.G.M.), with 3 years of experience, performed a targeted US examination and provided Breast Imaging Reporting and Data System (BI-RADS)^22^ assessments (1, negative; 2, benign; 3, probably benign; 4a, low suspicion; 4b, moderate suspicion; 4c, high suspicion; 5, highly suspicious for malignancy) of the lesions scanned on the in-house Philips HD11XE machine. All women with a mass scored BI-RADS ≥ 4a underwent US-guided core biopsy according to the standard of care at the hospital.

## RESULTS

The median age of the patients in the study was 46 years (range, 18 to 67 years). Of the 32 masses, two (6%) were malignant: a known cancer (BI-RADS 6 lesion) and one BI-RADS 5 mass, both invasive ductal carcinomas. The 12 other biopsied masses, assessed as BI- RADS 4a or 4b, proved to be benign and included seven fibroadenomas, two simple cysts, one fibrocystic disease, one intraductal papilloma, and one phyllodes tumor. The size of the masses imaged ranged from 0.8 to 5 cm, with a median of 1.8 cm. Five lesions were not measured, because they were larger than the 5-cm transducer width.

**Fig 4:**
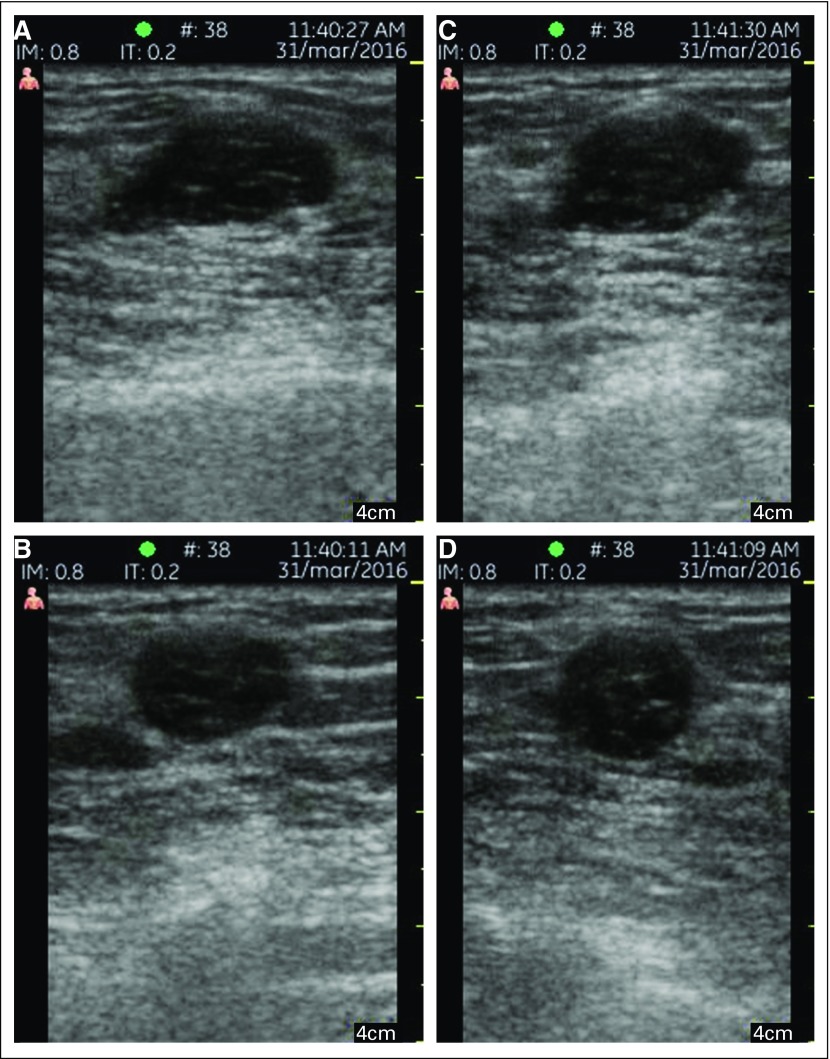
Images scanned with the GE Vscan Dual Probe on a woman with palpable lumps. Orthogonal images taken by (A, B) the trainee and (C, D) the radiologist.

The women were able to identify their palpable lumps for the trainees, who then acquired the appropriate images using the handheld US. Images scanned by a trainee versus a trained radiologist using the GE Vscan Dual Probe are shown in Figure 4. The triage-CADx software evaluated each image from the orthogonal pair separately, and it was found that taking the maximum score produced the best result for the triage-CADx system. This is consistent with radiologist BI-RADS assessment on the basis of the view that is most suspicious.

**Fig 5:**
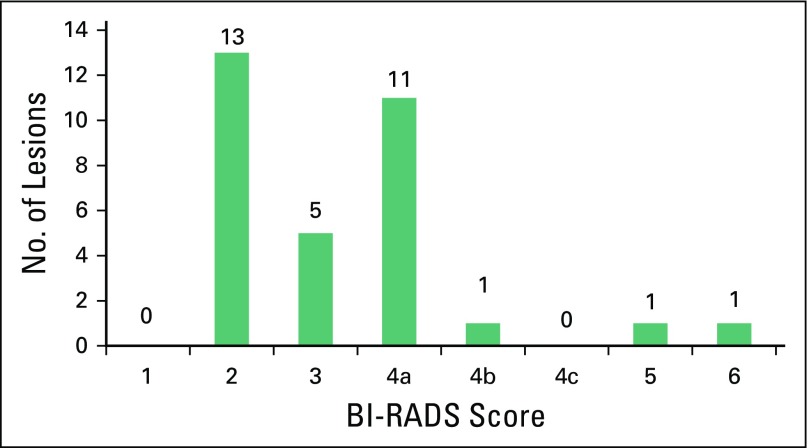
Radiologist-assessed Breast Imaging Reporting and Data System (BI-RADS) scores of lesions obtained during the study in Mexico. All lesions assessed as BI-RADS ≥ 4a are recommended for biopsy. The known cancer (BI-RADS 6) and one BI-RADS 5 lesion proved to be invasive cancers. All other lesions BI-RADS ≥ 4a sent for biopsy were found to be benign.

The distribution of BI-RADS scores for the lesions is shown in Figure 5. The radiologist assessed 13 masses as suspicious (BI-RADS ≥ 4a), five as BI-RADS 3, and 13 as BI-RADS 2. There was also one known cancer (BI-RADS 6). The known cancer (BI-RADS 6) and one BI-RADS 5 lesion proved to be invasive cancers, and 12 other lesions sent for biopsy were found to be benign.

**Fig 6:**
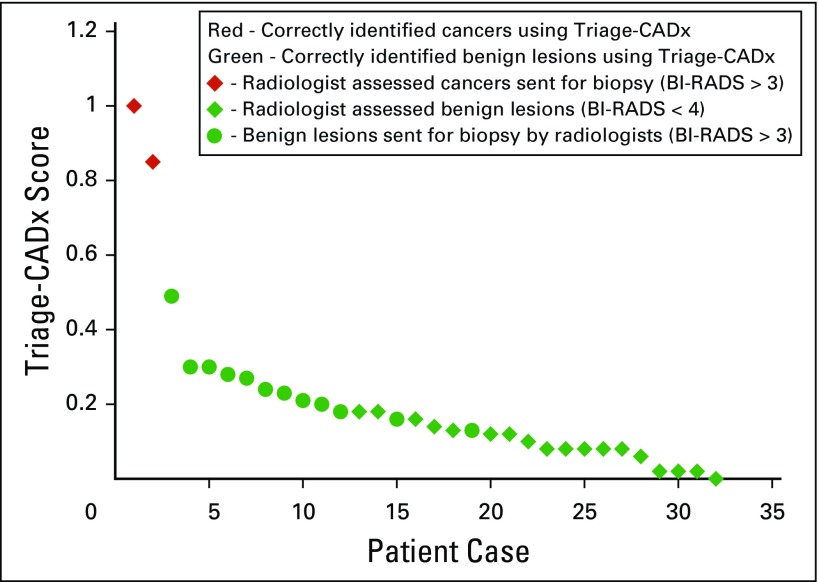
Triage–computer-assisted diagnosis (CADx) scores.

The triage-CADx system correctly classified all 13 BI-RADS 2 and five BI-RADS 3 lesions as benign. Figure 6 shows the scores from the triage-CADx system using the GE Vscan Dual Probe device operated by the trainees. These results are concordant with the scores on the basis of the GE Vscan Dual Probe images obtained by the radiologist. The GE Vscan Dual Probe images captured by a trainee or the radiologist resulted in the highest scores for the cancers (cases 28 and 27) followed by the benign lesions; all cancers were assigned to the red category (suspicious, recommended for biopsy), and all benign lesions (assessed as BI-RADS 4b, 4a, 3, or 2) to the green category (benign, no further action necessary).

This study demonstrates that first-level health care workers are capable of acquiring images with a portable US machine comparable to those of a trained radiologist. The triage-CADx system was applied to both the trainee and radiologist images with 100% sensitivity (two of two), 100% specificity (30 of 30), and an AUC of 1.0, enabling triage-CADx to correctly identify 30 benign lesions and the two cancers.

## DISCUSSION

At this site, the average wait for a diagnostic US is 9 months because of limited available trained radiologists. Many of the participants traveled up to 6 hours by bus to participate in the study, corroborating the need for a simple accessible local means of triage. This approach could potentially be applied among women participating in military deployment, remote scientific exploration, or space flights, where the ability to triage a palpable lump on site could conserve much-needed resources.

In this study of 32 women with 32 palpable breast masses, inexperienced operators were able to obtain diagnostic images using low-cost portable US comparable to those acquired by a trained radiologist. On the basis of those images, triage-CADx software accurately classified all masses, including two malignancies.

Our previous results using high-end US equipment^21^ showed a hypothetic reduction in benign biopsies by 40% while maintaining a sensitivity of 92%. The greatest improvement in specificity came from downgrading masses assessed as low suspicion (BI-RADS 4a) by breast-imaging specialist radiologists. In the validation study at Keck Medical Center of University of Southern California and Harbor–University of California Los Angeles Medical Center, experienced breast-imaging radiologists obtained orthogonal views of palpable lesions using high-end US devices. Evaluation of 156 women with 170 palpable masses demonstrated that triage-CADx achieved a sensitivity of 100% and specificity of 85%,^21^ corresponding to a reduction in the number of benign biopsies of 69%.

Although the number of masses in this preliminary study was small, these initial results are promising, showing a perfect separation of malignant and benign lesions, resulting in a sensitivity of 100%, specificity of 100%, and AUC of 1.0. In practice and with a larger number of patient cases, we expect these numbers to change. It is important to note that the triage-CADx system in this study was trained and validated using high-end cart-based US systems with transducer frequencies ≥ 12 MHz as recommended for breast imaging. Although the portable study machine (GE Vscan Dual Probe) acquired images at a frequency of 8 MHz, the triage-CADx system was still able to deliver excellent results on these images.

Phase II of this project is under way and includes the same study design using the GE Vscan (General Electric Medical Systems, Waukesha, WI) and a much larger number of participants; it will compare AUC results of the triage system with the standard-of-care radiologist performance on high-end US equipment with histopathologic confirmation.

This approach is not intended for screening but rather for triage in the field of palpable breast lumps to better allocate scarce health care resources. This small series demonstrates the ability of first-level health care workers with minimal training to locate and capture US images of palpable lumps equivalent to those of trained radiologists. We also confirmed that the images from a portable handheld US device were adequate for analysis by the triage-CADx system. This ability will be further confirmed in our ongoing larger study.

Although the device used in this study is more expensive than most LMIC settings can afford, lower-cost devices are becoming more available, and we have shown that our artificial intelligence platform generalizes across different hardware devices so that it can be applied to new hardware platforms as they come to market.

We have demonstrated that a minimally trained health care worker can effectively capture images of palpable breast lumps that are equivalent to those obtained by trained radiologists and are suitable for triage-CADx analysis. In addition, a triage system trained to distinguish suspicious and benign breast lesions can be used with a low-cost portable US system. We are currently enrolling women in a larger prospective study in Jalisco and Tijuana, Mexico, with the goal of demonstrating that health care workers without radiology training will be able to use this device in nonurban areas to accurately distinguish palpable lumps that require biopsy from those that do not.
